# The hidden link: dysmenorrhea, emotion regulation, and attitudes toward marriage in female nursing students

**DOI:** 10.1186/s12912-024-02341-w

**Published:** 2024-10-08

**Authors:** Shaimaa Mohamed Amin, Mona Metwally El-Sayed, Ahmed Hashem El-Monshed, Mahmoud Abdelwahab Khedr, Mohamed Hussein Ramadan Atta

**Affiliations:** 1 Lecturer of Community Health Nursing Faculty of Nursing, Damanhour University, Damanhour City, Egypt; 2 Assistant Professor of Psychiatric and Mental Health Nursing, Faculty of Nursing, Alexandria University, Alexandria City, Egypt; 3 Department of Psychiatric and Mental Health Nursing, Faculty of Nursing, Mansoura University, Mansoura, Egypt; 4 Department of Nursing, College of Health and Sport Sciences, University of Bahrain, Manama, Bahrain

**Keywords:** Dysmenorrhea, Emotion regulation, Attitudes toward marriage, Female nursing students

## Abstract

**Background:**

Menstrual pain, or dysmenorrhea, can cause physical discomfort and mood swings, potentially impacting the attitudes of female nursing students toward marriage. Effective emotion regulation strategies are essential for managing stress and shaping their perspectives on marriage. This study sought to investigate the relationship between dysmenorrhea, emotional regulation skills, and attitudes toward marriage among female nursing students.

**Methods:**

A cross-sectional survey was conducted using a stratified sample of 504 female nursing students from four academic years. The study used a combination of the Working Ability, Location, Intensity, Days of Pain, Dysmenorrhea (WaLIDD) Questionnaire, the Marital Attitude Scale, and the Emotion Regulation Questionnaire to assess various factors among the participants. The Pearson correlation coefficient was utilized to examine the relationships among the three primary variables. Additionally, linear regression analysis was employed to forecast attitudes toward marriage based on factors such as dysmenorrhea, residence, family type, regularity of the menstrual cycle, and age at menarche.

**Results:**

The findings of the study are significant, revealing a negative correlation between dysmenorrhea and marital attitudes (*r* = -0.105, *p* = 0.019). Dysmenorrhea exhibited minimal and non-significant correlations with emotion regulation strategies, including cognitive reappraisal (*r* = -0.006, *p* = 0.898) and expressive suppression (*r* = 0.013, *p* = 0.774). In contrast, marital attitudes significantly influenced emotion regulation (β = -0.169, *p* < 0.001). Moderation analyses indicated significant effects of various factors on marital attitudes, including residence (β = -0.5136, *p* = 0.0478), family type (β = 5.9923, *p* = 0.0060), regularity of the menstrual cycle (β = 6.1262, *p* = 0.0014), and age at menarche (β = 1.5521, *p* = 0.0491).

**Conclusion:**

A significant negative correlation between dysmenorrhea and certain attitudes, specifically optimistic and realistic marital attitudes, was found. This indicates that higher levels of dysmenorrhea are associated with less favorable perspectives in these domains. Additionally, the study demonstrates that negative marital attitudes were linked to poorer emotional regulation. This suggests that individuals with more pessimistic views about marriage may face challenges in effectively managing their emotions. These results underscore the intricate connections between physical health, attitudes, and emotional well-being. They highlight the need to address dysmenorrhea within nursing education and practice, considering its broader psychological impact.

**Supplementary Information:**

The online version contains supplementary material available at 10.1186/s12912-024-02341-w.

## Introduction

Dysmenorrhea, also known as painful menstruation, is a common issue for many adolescent females [1]. It is a natural occurrence experienced by almost every woman during her reproductive years [2]. Symptoms of dysmenorrhea appear before or during the menstrual cycle and include painful or cramping sensations in the lower abdomen, often accompanied by additional symptoms such as fatigue, sweating, headaches, nausea, vomiting, and diarrhea [3]. Dysmenorrhea can be classified into two types: primary and secondary. Primary dysmenorrhea (PD) occurs when clinical evaluations reveal no palpable lesions in the pelvis and is typically caused by excessive or abnormal uterine contractions [4]. On the other hand, secondary dysmenorrhea (SD) arises from acquired lesions in the pelvis, including conditions such as endometriosis, chronic pelvic inflammation, uterine fibroids, and cervical stenosis [5].

In academic settings, many female students suffer from menstrual disorders, such as irregular menstrual cycles and discomfort due to menstruation, especially primary dysmenorrhea. This widespread and often debilitating condition significantly affects their daily lives and emotional well-being [6, 7]. A study at Assiut University in Egypt found a prevalence rate of 90.4% among nursing students [8]. Similarly, previous research conducted in Saudi Arabia found a high prevalence of dysmenorrhea among female students, with 70.6% reporting symptoms [9].

Dysmenorrhea can cause moderate discomfort for nursing students during their menstrual periods. It can also lead to significant functional limits for some students. Lower abdominal and back pains are strongly associated with decreased academic and social performance and reduced productivity [10, 11]. Nursing education is significantly challenged by dysmenorrhea due to its high prevalence, costly medications, and interference with students’ productivity [12]. Additionally, the heavy duties and responsibilities of nursing practices significantly impact females’ lives through factors such as long working hours, burnout, disaffection, and effects on family ties [13, 14]. Therefore, it’s important to recognize the potential negative impacts of dysmenorrhea on nursing students’ future reproductive health concerns and their attitudes toward marriage. Attitudes toward marriage can be categorized as pessimistic, optimistic, realistic, and idealistic and may be influenced by negative past experiences or societal influences [15, 16].

On one hand, an optimistic attitude views marriage as a source of lasting happiness and fulfillment. Optimists have faith in the power of love and commitment, and they expect challenges to be overcome with mutual effort and support. On the other hand, the realistic attitude strikes a balance by acknowledging the joys and difficulties of marriage. Realists understand that marriage requires hard work, compromise, and communication, and they approach it with practical expectations and a readiness to navigate its ups and downs. Lastly, an idealistic attitude envisions marriage as a perfect union where partners share an unbreakable bond and complete understanding. Idealists often hold high, sometimes unattainable, standards for their relationships, believing true love can conquer all obstacles [15, 16].

There have been limited studies on attitudes toward marriage, especially among nursing students [17]. Attitudes toward marriage in nursing students can influence their relationship behaviors and expectations. Factors such as menstrual health (including age at menarche, premenstrual syndrome, menstrual cycle irregularities, and dysmenorrhea) could play a role. Research suggests that negative menstrual attitudes in female nursing students may impact their quality of life and influence their views on relationships and marriage [5, 10]. Negative attitudes toward marriage have been linked to higher distress levels among spouses of depressed individuals, even after controlling for marital adjustment. Studies have shown that more embedded negative marriage attitudes predict increased relationship conflict, lower commitment, and less expectation of relationship success [16, 18].

Furthermore, sociocultural factors and place of residence significantly shape nursing students’ attitudes toward marriage, influenced by family traditions, religious beliefs, and societal norms [19]. Cultural values and standards have a significant impact on the attitudes of nursing students toward marriage. Studies showed that consanguineous marriages (CMs) were common in Arabic countries and influenced by cultural and familial expectations. Many students hold positive attitudes towards these unions due to perceived compatibility and the continuity of cultural values [19]. Additionally, research indicates that couples with positive attitudes toward marriage, effective communication skills, and emotional regulation (ER) abilities are likely to improve their emotional stability and enhance their sexual intimacy, ultimately leading to increased marital satisfaction in various situations [20].

Emotion regulation skills are important in shaping attitudes towards marriage. Effective emotion regulation strategies can improve emotional stability and marital satisfaction [21]. Gross’s model on emotion regulation emphasizes the significance of using adaptive strategies to manage emotional responses, especially in females dealing with menstrual dysphoria. This highlights the connection between emotion regulation and menstrual pain [20–22]. Research indicates that it’s crucial to explore the relationship between menstrual pain, emotion regulation, and psychological factors. This highlights the need for further studies on how emotion regulation strategies may impact attitudes towards marriage, especially in the context of managing stressful situations like dysmenorrhea [23–25].

Dysmenorrhea is a common issue among nursing students, affecting their daily functioning and emotional well-being and potentially influencing attitudes toward marriage. Further research in Egypt and the MENA region is necessary to explore the connection between dysmenorrhea, emotion regulation, and attitudes towards marriage. Understanding the relationship between these factors can provide valuable insights into female nursing students’ overall well-being and perceptions of life. This knowledge can help develop targeted interventions to support their emotional and reproductive health. Therefore, this study aimed to investigate the association between primary dysmenorrhea, emotion regulation, and attitudes toward marriage among female nursing students.

### Research question

How does the severity of dysmenorrhea symptoms correlate with the emotional regulation abilities of female nursing students, and how does this relationship influence their attitudes toward marriage?

## Methods

### Study design

A cross-sectional survey was utilized for this study, with adherence to the STROBE checklist.

### Setting

The research was conducted at the College of Nursing, Damanhour University, in El-Beheira Governorate, Egypt. It comprises nine specialized scientific departments covering various nursing disciplines. The college’s undergraduate and graduate programs follow the credit hours system, offering a structured framework for monitoring academic progress and facilitating comprehensive evaluations of educational outcomes.

### Sample size and study participants

The target group for this study consisted of female undergraduate nursing students. To be eligible to participate, students had to meet the following criteria: being single, Egyptian, currently enrolled in the College of Nursing for the 2023–2024 academic year, and expressing willingness to participate. Participants were required to provide a detailed medical history, which was reviewed for any diagnosed pelvic disorders. Exclusion criteria included any diagnosed pelvic disorders (e.g., endometriosis, fibroids, pelvic inflammatory disease), hormonal imbalances, or other medical conditions known to cause secondary dysmenorrhea.

The sample size was calculated using MedCalc Statistical Software version 19.1 (MedCalc Software by Ostend, Belgium) [26]. To ensure adequate power and precision, a Type I error (alpha) of 0.05 and a Type II error (beta) of 0.10 (corresponding to a power of 0.90) were employed. The null hypothesis value, representing the prevalence of primary dysmenorrhea from a previous study, was set at 80% [25]. However, considering potential differences in population characteristics, the expected proportion of PD in the current study was conservatively estimated at 74%. Using these parameters, the sample size required to detect a significant difference was approximately 504 participants. This sample size ensures that the study is adequately powered to detect meaningful differences in the population mean with high confidence.

### Sampling and recruitment

A stratified sampling method was used to ensure a representative sample from each academic year. Within each academic year, students were further divided by their semester, and a proportional allocation strategy was used to select participants from each group. The Faculty of Nursing’s Student Affairs Department reported a total of 2,623 female undergraduate students enrolled during the academic year, distributed across the four academic years as follows: 742 in the first year, 687 in the second year, 557 in the third year, and 637 in the fourth year.

**Fig. 1:**
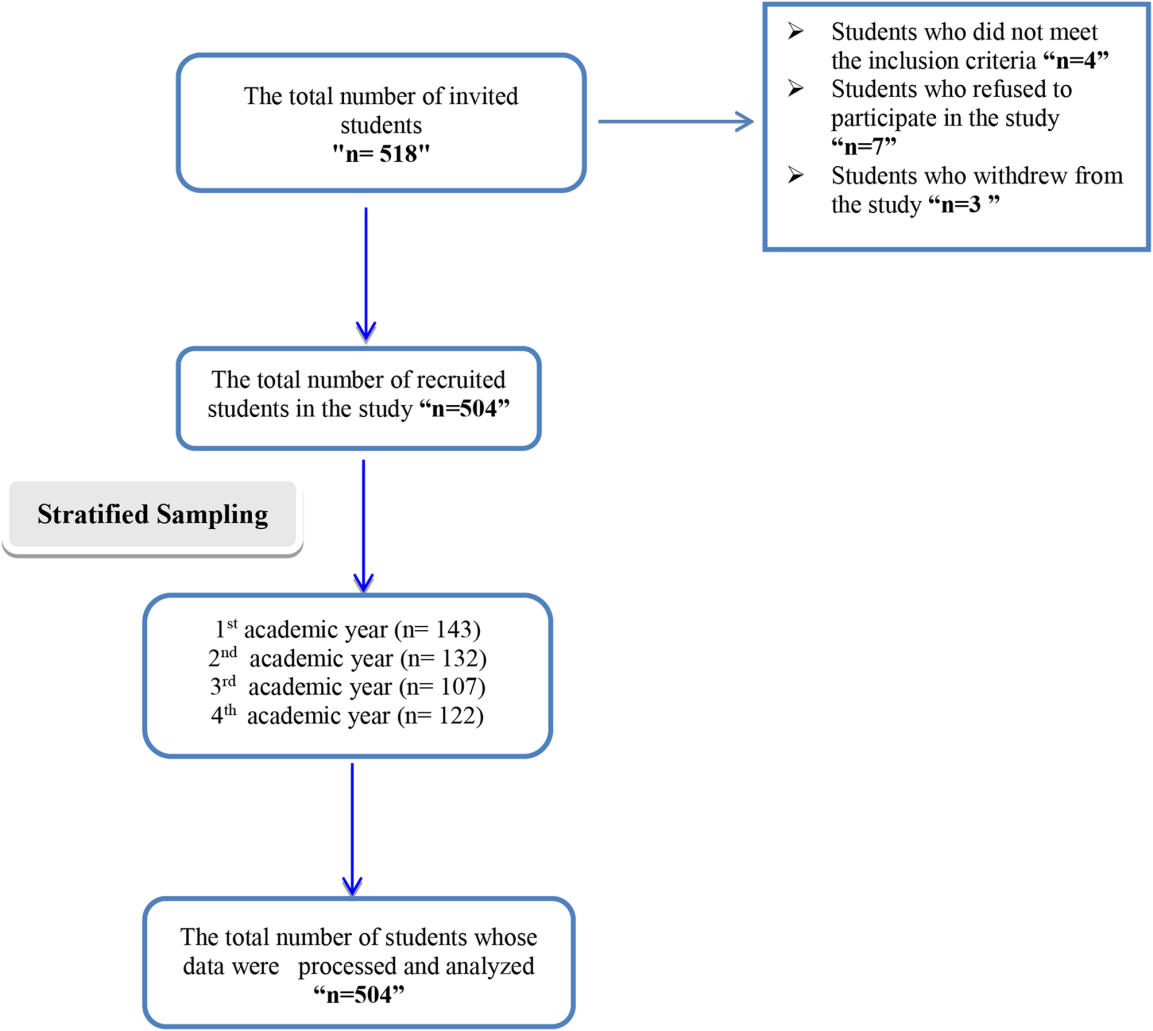
Flow chart of participants’ recruitment

A total of 518 female students were invited to participate in the study. However, 14 students withdrew from the study, with four being ineligible, seven refusing to participate, and three withdrawing, resulting in a response rate of 97.2%. A final sample of 504 students was obtained from the four academic semesters using a stratified randomization technique and proportional allocation method to ensure representativeness. The sample included 143 students from the first academic year, 132 from the second year, 107 from the third year, and 122 from the fourth year (Fig. 1).

### Measurements of interest

#### Socio-demographic and menstrual form

The Socio-demographic and Menstrual Form collects information about the participant’s age, academic year, occupation, place of residence, family type, and family income. It also gathers details about the participant’s menstrual history, including the age of the first period, duration and regularity of menstruation, previous use of pain relief methods, and family history of primary dysmenorrhea. The form also covers lifestyle factors such as exercise habits, dietary regimen, sleep patterns, smoking behavior, exposure to passive smoking, and frequency of medical checkups.

#### Working ability, location, intensity, days of pain, dysmenorrhea (WaLIDD) questionnaire

Teherán et al. (2018) created the WaLIDD questionnaire to evaluate dysmenorrhea, a common issue affecting many young women’s quality of life. The questionnaire covers five main areas: working ability, pain location, intensity, days of pain, and severity [27]. It is a self-rated Likert-type scale, ranging from 0 for none to 3 for severe. The interpretation of the WaLIDD questionnaire is as follows: 0 = No Dysmenorrhea; 1–4 = Mild Dysmenorrhea; 5–7 = Moderate Dysmenorrhea; 8–12 = Severe Dysmenorrhea. The WaLIDD score demonstrated acceptable internal consistency with a Cronbach’s alpha of 0.723 [27]. In this study, the Arabic version of the questionnaire showed excellent internal consistency with a Cronbach’s alpha coefficient of 0.89.

#### Martial attitude scale (MAS)

The Marriage Attitude Scale (MAS), developed by Braaten and Rosén in 1998, is a self-rated Likert scale of 23 questions [28]. It is designed to measure individuals’ attitudes toward marriage across various dimensions. These dimensions include pessimistic, optimistic, realistic, and romantic attitudes toward marriage. The scale uses a scoring system where Strongly Agree is scored as 0 and Strongly Disagree as 3. The individual item scores are then summed up to obtain a total score ranging from 23 to 92, with higher scores indicating a more positive attitude toward marriage. The original version of the MAS demonstrated good internal reliability with a Cronbach’s alpha of 0.82 and validity, as indicated by moderate correlations with other measures of marital attitudes (*r* = 0.77). In an Iranian study, the questionnaire’s content and convergent validity were confirmed, with a reliability coefficient of 0.76 using Cronbach’s alpha, indicating desirable reliability. Furthermore, the scale was translated into Arabic, and the Arabic version in a subsequent study showed a Cronbach’s alpha coefficient of 0.88, indicating excellent internal consistency.

#### Emotion regulation questionnaire (ERQ)

The Emotion Regulation Questionnaire (ERQ) is a self-report Likert-type scale comprising ten items. It measures individuals’ tendency to use two emotion regulation strategies: Cognitive Reappraisal and Expressive Suppression [29]. Cognitive reappraisal involves changing one’s interpretation of situations to regulate emotions (for example, “I control my emotions by changing the way I think about the situation I’m in”). On the other hand, Expressive Suppression involves holding back the outward expression of emotions (for example, “I control my emotions by not expressing them”). Respondents evaluate their agreement with each statement on a 7-point Likert scale, ranging from 1 (strongly disagree) to 7 (strongly agree), with higher scores indicating greater use of the respective strategy. The Arabic version of ERQ had a Cronbach’s alpha of 0.82 for the cognitive reappraisal subscale and 0.85 for the emotional suppression subscale [30]. In the current study, Cronbach’s alpha coefficient was calculated to be 0.86 for the cognitive reappraisal subscale and 0.87 for the emotional suppression subscale, indicating that the ERQ scale has high internal consistency reliability.

### Study procedures

#### Ethical considerations

The study received approval from the Research Ethics Committee of the Faculty of Nursing at Damanhur University, Egypt (under reference number 99). The study strictly followed the ethical standards outlined in the Helsinki Declaration to ensure the integrity of the research. All participants were provided comprehensive information about the study’s goals and methods. Written informed consent was obtained from all participants. The research also guaranteed voluntary participation, anonymity, and confidentiality. Participants were explicitly informed of their right to withdraw from the study without facing any consequences, reinforcing the study’s ethical considerations.

#### Pilot study

A pilot study was conducted with 50 female students randomly selected from the academic affairs list using the Research Randomizer tool, version 0.4. The aim was to assess the clarity and relevance of our study instruments, including the Working Ability, Location, Intensity, Days of Pain, Dysmenorrhea Questionnaire, Marital Attitude Scale, and Emotion Regulation Questionnaire. The findings indicated that all questions were clear and understandable, with no reported confusion. Additionally, the questions were appropriate for capturing the participants’ experiences with dysmenorrhea, their emotion regulation strategies, and their attitudes toward marriage, with no changes suggested. Participants also found the length and sequence of the questionnaires to be manageable and logical, and there were no technical issues in the random selection process. Given these results, we concluded that our study tools were well-suited for the main study and required no modifications. This pilot study confirmed our instruments’ adequacy and reliability, enhancing our research’s validity.

#### Data collection

Data collection for this study was conducted from April to May 2024. The process was initiated after obtaining the necessary permissions and acquiring Excel sheets containing details of all undergraduate students from the academic affairs department. The participants were selected using a random generator 0.4. This process was repeated until the required number of students was selected from each academic year. Before the commencement of data collection, the researchers explained the study’s aims to each student, emphasizing the voluntary nature of their participation. Written informed consent was obtained from each participant as a mandatory step before their involvement in the study. The researchers also reassured the participants about the confidentiality of their responses to foster trust in the research process. The questionnaires were distributed to students in quiet settings, including vacant lecture halls and libraries. Distribution occurred between 9 am and 2 pm from Saturday to Thursday. On average, participants spent 15 to 20 min completing each questionnaire.

### Data analysis

Version 29 of SPSS was employed to conduct statistical analyses of the collected data. Descriptive statistics, including percentages and frequency counts, were utilized to summarize all categorical variables, providing a clear overview of the distribution of responses within each category. For continuous variables, mean and standard deviation (SD) were calculated to characterize the central tendency and variability of the data. The Pearson correlation coefficient (r) explored associations between continuous variables. Linear regression analysis was conducted to examine how dysmenorrhea influences attitudes toward marriage. Additionally, moderation analysis was performed using the PROCESS macro for SPSS to assess the interaction effects [31]. Finally, statistical significance was determined using a conventional alpha (α) level threshold set at 0.05. Results with p-values less than 0.05 were considered statistically significant, indicating a low likelihood of observing the obtained results by chance alone.

## Results

Table 1 shows that 37.5% of the sample’s participants were between 20 and 22 years old. The majority resided in rural areas (61.7%). A notable proportion were not working (76.4%), while 23.6% reported being employed. Regarding family structure, nuclear families constituted the majority (63.5%), and 52.6% reported having enough monthly income. Most participants reported not practicing regular exercises (84.9%), more than half reported following a healthy diet (57.5%), and 42.5% reported consuming unhealthy foods. Regarding sleeping patterns, the majority reported having a regular sleeping pattern (55.4%), while 44.6% reported experiencing insomnia. A considerable proportion of participants reported exposure to passive smoking (60.7%). A small percentage of participants reported undergoing periodic medical checkups every six months (2.2%), while the majority did not (97.8%).

**Table 1:** Socio-demographic characteristics of the study participants (*N* = 504)

Socio-demographic Factors	*N*	%
**Age (in Years):**		
> 20	174	34.5
20 - > 22	189	37.5
≥ 22	141	28.0
**Occupation**:		
Working	119	23.6
Not Working	385	76.4
**Residence**:		
Rural	311	61.7
Urban	193	38.3
**Type of Family**:		
Nuclear	320	63.5
Extended	184	36.5
**Family Income**:		
Enough	265	52.6
Enough & save	96	19.0
Not Enough	143	28.4
**Lifestyle Habits**		
**Practicing Regular Exercises**		
No	428	84.9
Yes	76	15.1
**Dietary Regimen**:		
Healthy Diet	290	57.5
Unhealthy Foods	214	42.5
**Sleeping Pattern**:		
Normal	279	55.4
Insomnia	225	44.6
**Exposure to Passive Smoking**:		
No	198	39.3
Yes	306	60.7
**Periodic Medical Checkup Every 6 Months**:		
No	493	97.8
Yes	11	2.2

Table 2 outlines the menstrual history of the study participants. The mean age of menarche was 12.67 years. On average, participants reported a menstrual duration of 5.89 days. Regular menstruation was reported by 57.3% of participants, while 42.7% reported irregular patterns. The most common pain relief approach was pharmacological use on one’s own (46.0%), followed by non-pharmacological use (22.4%) and pharmacological use as per doctor’s orders (3.6%). Approximately half of the participants reported a family history of primary dysmenorrhea (46.0%). Regarding the severity of dysmenorrhea, 62% experienced severe symptoms, 25.5% experienced moderate symptoms, and 4% experienced mild symptoms. The average severity score was 7.45, with a standard deviation of 2.92.

**Table 2:** Menstrual history of the study participants (*N* = 504)

Reproductive History	*N*	%
Age of Menarche (in Years):	M (SD) = 12.67 (1.29)	
Duration of Menstruation (in Days):	M (SD) = 5.89 (1.18)	
Regularity of Menstruation:		
Regular	289	57.3
Irregular	215	42.7
Previous use of pain relief method:		
No Use of pain relief	141	28.0
Pharmacological use as doctor’s order	18	3.6
Pharmacological use as on her own	232	46.0
Non-pharmacological use	113	22.4
Family history of primary dysmenorrhea:		
No	272	54.0
Yes	232	46.0
Dysmenorrhea:	M (SD) = 7.45 (2.92)	
No	43	8.5
Mild	20	4
Moderate	129	25.5

*M* = Mean, *SD* = Standard Deviation

Table 3 presents the descriptive statistics of the study measures among the participants. The mean total score for Marital Attitude was 48.42 (SD = 7.97). The sub-dimensions were as follows: Pessimistic Attitude (M = 13.25, SD = 3.26), Optimistic Attitude (M = 10.30, SD = 2.50), Realistic Attitude (M = 19.94, SD = 3.83), and Idealistic Attitude (M = 4.93, SD = 1.40). The mean total score for Emotion Regulation was 47.76 (SD = 8.46). The sub-dimensions were Cognitive Reappraisal (M = 28.67, SD = 5.51) and Expressive Suppression (M = 19.09, SD = 4.24).

**Table 3:** Descriptive statistics of the study measures (*N* = 504)

Study Measures	M	SD	Range	Minimum	Maximum
Marital Attitude (Total)	48.42	7.97	57	19	76
Pessimistic Attitude	13.25	3.26	24	0	24
Optimistic Attitude	10.30	2.50	15	3	18
Realistic Attitude	19.94	3.83	28	5	33
Idealistic Attitude	4.93	1.40	7	2	9
Emotion Regulation (Total)	47.76	8.46	52	18	70
Cognitive Reappraisal	28.67	5.51	35	7	42
Expressive Suppression	19.09	4.24	24	4	28

*M* = Mean, *SD* = Standard Deviation

Table 4 displays the correlation coefficients among dysmenorrhea, marital attitudes, and emotion regulation strategies. Dysmenorrhea was negatively correlated with optimistic attitudes (*r*= -0.088, *p* = 0.048), realistic attitudes (*r*= -0.122, *p* = 0.006), and overall marital attitudes (*r* = -0.105, *p* = 0.019). Correlations with pessimistic attitudes (*r*= -0.040, *p* = 0.370) and idealistic attitudes (*r*= -0.010, *p* = 0.817) were weak and non-significant. Dysmenorrhea showed minimal correlations with cognitive reappraisal (*r*= -0.006, *p* = 0.898) and expressive suppression (*r* = 0.013, *p* = 0.774). The overall emotion regulation score had a negligible correlation with dysmenorrhea (*r* = 0.003, *p* = 0.952). Marital attitudes were significantly positively correlated with cognitive reappraisal (*r* = -0.163, *p* < 0.001), expressive suppression (*r*= -0.125, *p* = 0.005), and the overall emotion regulation score (*r*= -0.169, *p* < 0.001).

**Table 4:** Correlation coefficient analysis between the study measures (*N* = 504)

Study Measures		1	2	3	4	5	6	7	8	9
Dysmenorrhea (1)	r	1								
	P									
Pessimistic Attitude (2)	r	-0.040	1							
	P	0.370								
Optimistic Attitude (3)	r	-0.088*	0.095*	1						
	P	0.048	0.034							
Realistic Attitude (4)	r	-0.122**	0.304**	0.812**	1					
	P	0.006	< 0.001	< 0.001						
Idealistic Attitude (5)	r	-0.010	0.021	0.328**	0.254**	1				
	P	0.817	0.636	< 0.001	< 0.001					
Marital Attitude Total (6)	r	-0.105*	0.589**	0.801**	0.905**	0.410**	1			
	P	0.019	< 0.001	< 0.001	< 0.001	< 0.001				
Cognitive Reappraisal (7)	r	-0.006	-0.043	-0.180**	-0.148**	-0.099*	-0.163**	1		
	P	0.898	0.338	< 0.001	< 0.001	0.026	< 0.001			
Expressive Suppression (8)	r	0.013	-0.019	-0.114*	-0.127**	-0.116**	-0.125**	0.496**	1	
	P	0.774	0.668	0.010	0.004	0.009	0.005	< 0.001		
Emotion Regulation Total (9)	r	0.003	-0.037	-0.175**	-0.160**	-0.123**	-0.169**	0.900**	0.825**	1
	P	0.952	0.401	< 0.001	< 0.001	0.006	< 0.001	< 0.001	< 0.001	

*r* = Pearson Correlation

*Correlation is significant at the 0.05 level (2-tailed)

**Correlation is significant at the 0.01 level (2-tailed)

Table 5 presents the results of four regression analyses examining the relationships between dysmenorrhea and various attitudes and the relationship between marital attitude and emotion regulation. Dysmenorrhea had negative effects on optimistic attitude (β= -0.08, *p* = 0.04), realistic attitude (β= -0.12, *p* = 0.006), and marital attitude (β= -0.10, *p* = 0.019). Marital attitude had a negative effect on emotion regulation (β = -0.16, *p* < 0.001).

**Table 5:** Summary of regression analyses (*N* = 504)

	B	SE	ß	t	*P*	95% LLCI	95% ULCI
Dysmenorrhea	-0.07	0.03	-0.08	-1.984	0.048	-0.150	-0.001
*R*^*2*^ = 0.008 (*Adj R*^*2*^ = 0.006), *F-*change = 3.935, *P* = 0.048, Dependent variable: Optimistic Attitude							
Dysmenorrhea	-0.16	0.05	-0.12	-2.763	0.006	-0.275	-0.046
*R*^*2*^ = 0.015 (*Adj R*^*2*^ = 0.013), *F-*change = 7.635, *P* = 0.006, Dependent variable: Realistic Attitude							
Dysmenorrhea	-0.28	0.12	-0.10	-2.359	0.019	-0.524	-0.048
*R*^*2*^ = 0.011 (*Adj R*^*2*^ = 0.009), *F-*change = 5.567, *P* = 0.019, Dependent variable: Marital Attitude							
Marital Attitude	-0.17	0.04	-0.16	-3.836	< 0.001	-0.271	-0.087
*R*^*2*^ = 0.028 (*Adj R*^*2*^ = 0.027), *F-*change = 14.717, *P* < 0.001, Dependent variable: Emotion Regulation							

Significant difference compared to the reference category ≤ 0.001

*B* = Coefficient, *SE* = standard error, *ß* = standardized coefficient beta; *t* = Student t test for linear regression

95% CI for difference 95% Confidence Interval for Difference

Table 6 presents the results of moderation analyses exploring how various factors (residence, family type, menstrual cycle regularity, and age at menarche) influence the relationship between dysmenorrhea and marital attitudes among female nursing students. The analysis revealed a significant interaction between dysmenorrhea and residence (β= -0.51, *p* = 0.0478), indicating that the relationship between dysmenorrhea and marital attitudes varies based on the type of residence. Specifically, for participants residing in urban areas, higher levels of dysmenorrhea were associated with lower marital attitudes, suggesting that urban living may exacerbate the negative impact of dysmenorrhea on perceptions of marriage, potentially due to differing social or environmental stressors.

**Table 6:** Moderation analysis of various moderators on the Relationship between Dysmenorrhea and Marital attitudes (*N* = 504)

Moderator	β	SE	t	*p*-value	95% LLCI	95% ULCI
**Residence**						
Dysmenorrhea	-0.01	0.14	-0.1222	0.9028	-0.3094	0.2731
Residence	1.07	2.14	0.4994	0.6177	-3.1473	5.2925
Dysmenorrhea × Residence	-0.51	0.25	-1.9836	0.0478	-1.0223	-0.0049
R²= 0.0492, F = 8.6163, df1 = 3, df2 = 050, p (Model) = 0.0000, R² Change = 0.0075, F (Interaction)= 3.9348, p (Interaction) = 0.0478.						
**Family Type**						
Dysmenorrhea	-0.23	0.13	-1.7676	0.0777	-0.4948	0.0261
Family Type	5.99	2.17	2.7612	0.0060	1.7285	10.2561
Dysmenorrhea × Family Type	-0.17	0.27	-0.6483	0.5171	-0.7232	0.3643
R²= 0.0910, F = 16.6914, df1 = 3, df2 = 050, p (Model) = 0.0000, R² Change = 0.0008, F (Interaction)= 0.4203, p (Interaction) = 0.5171.						
**Regularity of Menstrual Cycle**						
Dysmenorrhea	-0.07	0.16	-0.4605	0.6454	-0.4025	0.2496
Regularity of Menstrual Cycle	6.12	1.90	3.2171	0.0014	2.3848	9.8675
Dysmenorrhea × Regularity of Menstrual Cycle	-0.38	0.23	-1.6167	0.1066	-0.8498	0.0826
R²= 0.0568, F = 10.0458, df1 = 3, df2 = 050, p (Model) = 0.0000, R² Change = 0.0049, F (Interaction)= 2.6138, p (Interaction) = 0.1066.						
**Age of Menarche**						
Dysmenorrhea	1.15	1.22	0.9422	0.3465	-1.2506	3.5552
Age of Menarche	1.55	0.78	1.9727	0.0491	0.0063	3.0979
Dysmenorrhea × Age of Menarche	-0.11	0.09	-1.1612	0.2461	-0.3017	0.0775
R²= 0.0263, F = 4.4971, df1 = 3, df2 = 050, p (Model) = 0.0040, R² Change = 0.0026, F (Interaction)= 1.3485, p (Interaction) = 0.2461.						
**Emotional Regulation Moderation**						
Dysmenorrhea	-0.239	0.125	-1.915	0.056	-0.485	0.006
Emotion Regulation (ER)	-0.158	0.041	-3.842	< 0.001	-0.239	-0.077
Dysmenorrhea × ER (Interaction)	-0.021	0.017	-1.219	0.224	-0.054	0.013
R²= 0.0422, F = 7.3431, df1 = 3, df2 = 050, p (Model) = 0.0001, R² Change = 0.0028, F (Interaction)= 1.4848, p (Interaction) = 0.2236.						

Significant difference compared to the reference category ≤ 0.001

*B* = Coefficient, *SE* = standard error, *ß* = standardized coefficient beta; *t* = Student t test for linear regression

95% CI for difference 95% Confidence Interval for Difference

In addition to this interaction, several main effects were identified. The main effect of family type was significant (β = 5.99, *p* = 0.0060), with participants from extended families reporting higher marital attitudes than those from nuclear families. However, the interaction between dysmenorrhea and family type was not significant, indicating that family structure does not significantly modify the relationship between dysmenorrhea and marital attitudes. The main effect of menstrual cycle regularity was also substantial (β = 6.12, *p* = 0.0014), demonstrating that regular menstrual cycles were associated with more positive marital attitudes, though this factor did not significantly moderate the dysmenorrhea-marital attitude relationship. Additionally, the age at menarche showed a significant main effect (β = 1.55, *p* = 0.0491), with older age at menarche linked to more favorable marital attitudes. However, similar to family type and menstrual cycle regularity, age at menarche did not significantly moderate the impact of dysmenorrhea on matrimonial attitudes. In summary, the findings suggest that while residence acts as a significant moderator in the relationship between dysmenorrhea and marital attitudes, other factors such as family type, menstrual cycle regularity, and age at menarche influence marital attitudes independently without moderating the relationship between dysmenorrhea and marital attitudes.

Additionally, the moderation analysis involving ER as a moderator in the relationship between Dysmenorrhea and Marital Attitudes revealed that while ER significantly impacts Marital Attitudes (β = -0.158, *p* < 0.001), it does not significantly moderate the relationship between Dysmenorrhea and Marital Attitudes (interaction term β = -0.021, *p* = 0.224). The model explains a modest 4.22% of the variance in Marital Attitudes, with the interaction term contributing only a small fraction of this variance. These findings suggest that while ER has a direct effect on Marital Attitudes, it does not alter the effect of Dysmenorrhea on Marital Attitudes in this sample.

## Discussion

The current study aimed to investigate the relationship between primary dysmenorrhea, emotion regulation, and attitudes toward marriage among female nursing students. It was found that female nursing students reported moderately positive attitudes toward marriage, with higher scores on realistic and optimistic attitudes than pessimistic and idealistic ones. This suggests that, on average, the participants held a balanced perspective on marriage without leaning too strongly towards either optimistic or pessimistic views. This pattern of marital attitudes among female nursing students is noteworthy, as it provides insight into how they may approach and navigate their interpersonal relationships. The moderate, realistic outlook could serve as a protective factor, helping them to navigate the complexities of marriage and relationships.

The relatively lower levels of pessimistic attitude indicate that the nursing students did not tend to hold an excessively cynical or pessimistic view of marriage. This is a positive finding, as a pessimistic mindset could undermine their ability to form and maintain healthy personal and professional relationships [32]. Maintaining a balanced perspective on the challenges of marriage while not succumbing to a purely negative outlook can help nursing students navigate their personal lives. Consistent with current study results, Russell et al. (2023) reported that nursing students expressed optimistic views about marriage and recognized the importance of addressing this in their future nursing practice. However, they also acknowledged that they would benefit from receiving foundational education and training to feel adequately prepared to handle these matters [33].

The participants reported a moderate ability to manage their emotions regarding emotion regulation. The higher score on cognitive reappraisal compared to expressive suppression indicates that the students were more likely to regulate their emotions by reframing situations in a more positive light rather than suppressing the outward expression of their feelings. This preference for cognitive reappraisal as an emotion regulation strategy aligns with research indicating that it is generally considered a more adaptive and practical approach compared to expressive suppression. Cognitive reappraisal has been associated with better psychological well-being, allowing individuals to modify their emotional responses early before the emotion is fully experienced and expressed [34].

In the context of nursing students, the ability to effectively regulate emotions through cognitive reappraisal may be particularly beneficial. Nursing is a highly demanding profession that often requires managing intense emotional situations, such as dealing with patient suffering, death, and other stressful events [35]. Nursing students’ tendency to use cognitive reappraisal may help them cope better with the emotional challenges they face in their personal and professional lives. This pattern of ER is consistent with research showing that negative feelings like depression, anxiety, and stress are closely linked to the severity and frequency of dysmenorrhea symptoms in nursing students [32, 36]. The ability to cognitively reappraise challenging situations may be a protective factor against the negative impact of dysmenorrhea on well-being and academic performance [37].

The correlation analysis of the current study reveals that severe levels of dysmenorrhea were associated with lower levels of positive marital attitudes. The negative correlation between dysmenorrhea and marital attitude may be explained by the impact of painful menstruation on the overall well-being and functioning of these students. This finding aligns with previous research indicating that many nursing students view menstruation negatively as limiting and debilitating, which can exacerbate their dysmenorrhea symptoms [32, 38]. Studies have shown that dysmenorrhea can lead to physical discomfort, mood changes, and limitations in daily activities, all of which may influence how these females perceive and approach the prospect of marriage [7, 39]. The stress and disruption caused by dysmenorrhea may contribute to a more pessimistic outlook on intimate relationships.

Interestingly, the study analysis found that dysmenorrhea has a limited impact on emotion regulation strategies. This suggests that, at least in this population, the experience of dysmenorrhea may not have a substantial effect on how individuals regulate their emotions. These results were contrary to those in previous research, which have indicated that negative feelings like depression, anxiety, and stress are closely linked to the severity and frequency of dysmenorrhea symptoms [39, 40]. Also, it was previously found that dysmenorrhea can have emotional impacts, with cognitive-behavioral therapy shown to be effective at reducing depression as a symptom [23], with a significant role of emotional support with nursing students [41]. The results also revealed that more negative marital attitudes are associated with poorer emotion regulation. A pessimistic outlook on marital relationships may contribute to heightened emotional distress, making it more challenging for individuals to manage their emotions effectively. Consistent with the current results, a recent study reported that attitude to marriage or marital dissatisfaction is a significant contributing factor to instability and emotional problems among nursing professionals [42].

The moderation analysis of the current results illustrated that the negative association between dysmenorrhea and marital attitudes was more pronounced for participants from urban areas compared to those from rural areas. This finding suggests that the broader social and cultural context may influence the impact of dysmenorrhea on marital attitudes. This finding aligns with existing literature suggesting that urban living conditions may exacerbate the psychological and social stressors associated with dysmenorrhea, potentially leading to a more negative outlook on relationships [43]. Additionally, the analysis highlighted that participants from extended families reported more positive attitudes than those from nuclear families. This suggests that family dynamics and support systems may be crucial in shaping attitudes toward marriage. Extended families often provide a broader support network, which could positively influence individual perspectives on marital relationships [44].

The regularity of the menstrual cycle also emerged as a significant main effect, suggesting that students with more regular cycles tend to have higher marital attitudes. Irregular menstrual cycles have been associated with increased psychological distress and reduced quality of life, which may, in turn, affect marital attitudes [45]. Moreover, the results indicate that students who experienced menarche at an older age tend to report higher marital attitudes. This finding could be related to the potential psychological and social implications of earlier versus later pubertal development. Earlier menarche has been linked to an increased risk of mental health issues and interpersonal difficulties, which may negatively impact marital attitudes. In contrast, later menarche may be associated with a more gradual and less disruptive transition to womanhood, potentially leading to more positive perceptions of marriage [46].

Moreover, The lack of significant moderation by emotional regulation (ER) in the relationship between dysmenorrhea and marital attitudes suggests that the influence of dysmenorrhea on marital attitudes remains consistent regardless of the levels of emotional regulation. One possible explanation for this finding is that emotional regulation may operate as a direct predictor of marital attitudes rather than as a conditional factor that alters the dysmenorrhea-marital attitudes relationship. Furthermore, the limited variance accounted for by the model suggests that other unexamined variables could have a more significant impact on marital attitudes. This highlights the intricate nature of psychological constructs and emphasizes the necessity for additional research to identify other moderators or mediators that could shed light on these dynamics.

## Limitations of the study

One primary limitation of this study is its cross-sectional design, which captures a snapshot of participants’ attitudes and experiences at a specific time. This design precludes establishing causal relationships between variables of interest, such as dysmenorrhea, emotion regulation (ER), and attitudes toward marriage. A longitudinal study design would better examine these factors’ dynamic relationships and potential changes over time. Additionally, the study was conducted within a single nursing college, which may limit the generalizability of the findings to the broader population of female nursing students in Egypt. Expanding the study to include participants from multiple nursing institutions nationwide could enhance the sample’s representativeness and strengthen the external validity of the results. Another potential limitation is the reliance on self-reported data, which may be subject to biases such as social desirability or recall bias. While validated and reliable instruments were employed, objective measures, such as menstrual pain assessments or physiological markers of emotion regulation, could provide a more comprehensive understanding of the phenomena under investigation.

### Nursing implications

Nursing programs should incorporate comprehensive menstrual health education, emotional regulation skills training, and marital and relationship education to help students manage dysmenorrhea, develop adaptive coping strategies, and foster a balanced perspective on intimate relationships. Fostering a supportive campus environment that destigmatizes menstrual health and provides access to relevant resources is also crucial. Academic members working with female nursing students should consider the potential impact of dysmenorrhea on their attitudes toward marriage. They need to provide support, education, and marital counseling to foster healthier attitudes toward marriage among this group.

## Recommendations

Based on our findings, we recommend including educational programs in nursing curricula to enhance students’ understanding and management of dysmenorrhea. Providing resources and training in emotion regulation techniques could help students better cope with the emotional effects of dysmenorrhea. Taking these practical steps can improve the well-being of nursing students and potentially enhance their professional performance and patient care skills. Future studies could explore the potential impact of interventions to improve emotion regulation strategies or address menstrual-related issues among female nursing students. Assessing the effectiveness of such interventions could help develop tailored support systems and educational programs to enhance this student population’s overall well-being and professional development. Further research with diverse populations and alternative methodological approaches could provide deeper insights into the potential moderating roles of ER.

## Conclusion

The findings of this study provide valuable insights into the connections between dysmenorrhea, emotion regulation, and attitudes toward marriage among female nursing students. The analysis revealed significant negative correlations between dysmenorrhea and certain attitudes, particularly optimistic and realistic marital attitudes. This suggests that higher levels of dysmenorrhea are associated with less favorable perspectives in these areas. Moreover, the study showed that negative marital attitudes were associated with poorer emotional regulation, indicating that individuals with more pessimistic views about marriage may struggle to manage their emotions effectively. These results underscore the complex links between physical health, attitudes, and emotional well-being, emphasizing the importance of addressing dysmenorrhea within nursing education and practice due to its broader psychological impact. It is important to acknowledge these relationships without exaggerating the findings, ensuring that recommendations are based on the observed data.

## Electronic supplementary material

Below is the link to the electronic supplementary material.

Supplementary Material 1

## Data Availability

The datasets generated during and analyzed during the current study are notpublicly available due to confidentiality agreements but are available uponreasonable request from the corresponding author.
